# Body Mass Index and Cancer Mortality Among Korean Older Middle-Aged Men: A Prospective Cohort Study

**DOI:** 10.1097/MD.0000000000003684

**Published:** 2016-05-27

**Authors:** Jae-Seok Hong, Sang-Wook Yi, Jee-Jeon Yi, Seri Hong, Heechoul Ohrr

**Affiliations:** From the Department of Healthcare Management, Cheongju University College of Health Sciences, Cheongju (J-SH); Department of Preventive Medicine and Public Health, Catholic Kwandong University College of Medicine, Gangneung (S-WY); Institute for Clinical and Translational Research (S-WY), Institute for Occupational and Environmental Health, Catholic Kwandong University, Gangneung (J-JY); Department of Preventive Medicine, Graduate School of Public Health, Yonsei University, Seoul (SH); Institute for Health Promotion, Graduate School of Public Health, Yonsei University, Seoul (HO); and Department of Preventive Medicine, Yonsei University College of Medicine (HO), Seoul, Republic of Korea.

## Abstract

The association of body mass index (BMI; kg/m^2^) with overall and site-specific cancer mortality in Asians is not well understood. A total of 113,478 men from the Korean Veterans Health Study who returned a postal survey in 2004 were followed up until 2010. The adjusted hazard ratios (HRs) of cancer mortality were calculated using a Cox model. During 6.4 years of follow-up, 3478 men died from cancer. A reverse J-curve association with a nadir at 25.0 to 27.4 kg/m^2^ was observed. Below 25 kg/m^2^, the HRs of death for each 5 kg/m^2^ decrease in BMI were 1.72 (95% confidence interval = 1.57–1.90) for overall cancer; 3.63 (2.57–5.12) for upper aerodigestive tract (UADT) cancers, including oral cavity and larynx [HR = 4.21 (2.18–8.12)] and esophagus [HR = 2.96 (1.82–4.81)] cancers; 1.52 (1.35–1.71) for non-UADT and non-lung cancers, including stomach [HR = 2.72 (2.13–3.48)] and large intestine [HR = 1.68 (1.20–2.36)] cancers; and 1.93 (1.59–2.34) for lung cancer. In the range of 25 to 47 kg/m^2^, the HRs for each 5 kg/m^2^ increase in BMI were 1.27 (1.03–1.56) for overall cancer mortality and 1.57 (1.02–2.43) for lung cancer mortality. In individuals <25 kg/m^2^, inverse associations with mortality from overall cancer and non-UADT and non-lung cancer were stronger in never-smokers than in current smokers. Both low and high BMI were strong predictors of mortality from overall and several site-specific cancers in Korean men. Further research is needed to evaluate whether interventions involving weight change (loss or gain) reduce the risk of cancer or improve the survival.

## Abstract 2

Supplemental Digital Content is available in the text

## INTRODUCTION

Body mass index (BMI, kg/m^2^) has been widely used to define obesity and thinness for clinical and public health interventions.^1^ Excess adiposity has been associated with elevated risk for several sex- and site-specific cancers.^2^ However, the evidence linking excess body weight to cancer has mainly been drawn from populations with a European origin.^2–4^ Few studies have examined these associations in Asian populations, which have different body fat distribution and composition, environmental risk factors (e.g., prevalence of hepatitis), and genetic backgrounds.^4–6^

U-curve associations of BMI with mortality have been commonly reported.^7–9^ A recent large prospective study indicated that Korean men with low normal weight (e.g., 18.5–22.9 kg/m^2^) had a higher risk of death than those with grade I obesity^9^; however, no information on cause-specific mortality was available. Compared with obesity, relatively few studies have explored the association of low BMI with mortality due to the low prevalence of low BMIs in European-origin populations.^10^ Because Asians generally have a thinner body shape than European-origin individuals, the association of lower BMI with mortality can be better explored in studies of Asian populations. Understanding the associations of both low and high BMI with mortality in various ethnic and regional groups will help develop strategies to mitigate the adverse health effects related to body weight in all ethnic and regional groups.^11^

The purpose of this study was to prospectively examine the association of BMI with overall and site-specific cancer mortality in Korean older middle-aged and elderly men recruited in 2004. Because smoking is known to increase the risk of many cancers, and is also associated with weight change, we additionally examined these associations stratified by smoking status to reduce the potential residual confounding related to smoking,^12^ as well as to examine the effect of smoking on the associations.

## METHODS

### Study Population

Of the 187,897 men in the Korean Veterans Health Study,^13,14^ 164,208 living veterans were identified in June 2004, with the exclusion of 23,689 individuals who were deceased or had emigrated. A postal survey was sent out on July 27, 2004, to which 117,609 veterans (71.6%) replied. Those with missing information on BMI (n = 3693) or whose residential status was uncertain after the initial survey (n = 438) were excluded. A total of 113,478 veterans were ultimately included in the analysis.

### Data Collection

Information on smoking, alcohol consumption, physical activity, height, weight, and income was collected from the survey. The BMI (kg/m^2^) was calculated from the self-reported weight in kilograms divided by the square of the self-reported height in meters. More details about the survey can be obtained elsewhere.^14,15^ The participants were assumed to have received the survey on August 1, 2004. Person-years were calculated from August 1, 2004 until the earliest of death, or the end of the study (December 31, 2010). Participants with any cancers diagnosed from January 1, 1992 to July 31, 2004 were identified through the National Cancer Incidence Database,^13^ and these were considered to be individuals with preexisting cancers. Participants were recorded as having certain medical conditions (such as viral hepatitis and liver diseases), if they visited a medical institution at least once for a given condition between January 1, 2000 and July 31, 2004.

Data on deaths and causes of death from August 1, 2004 to December 31, 2010 were confirmed by national death records from the National Statistical Office of Korea. Follow-up was performed through record linkage at the national level and was complete. *The International Classification of Diseases 10th Revision* (ICD-10) was used to classify the cancer deaths.^16^ The mortality outcomes and corresponding ICD-10 codes are shown in eTable 1.

### Statistical Analysis

BMI values were categorized into 7 groups (kg/m^2^: <18.5, 18.5–20.9, 21.0–22.9, 23.0–24.9, 25.0–27.4 [reference], 27.5–29.9, and ≥30) using cutoff points suggested by the World Health Organization.^1^ The reference BMI category was selected based on previous research in East Asian men showing that a BMI of approximately 25 to 27 kg/m^2^ was associated with the lowest risk of mortality.^8,17–19^ BMI was also used as a continuous variable to estimate the effect of each 5 kg/m^2^ increase or decrease on cancer mortality within the ranges of 12 to 24.9, 25 to 47, and 12 to 47 kg/m^2^.

Cox proportional hazards models were used to calculate hazard ratios (HRs) after adjustment for the following covariates: age at enrollment (continuous variable), smoking (never, past, current smoker, or missing information [n = 1147]), frequency of alcohol consumption (5 or more times/week, 1 to 4 times/week, <1 time/week, prior drinker [no alcohol for a year], never-drinker, or missing information [n = 1464]), physical activity (10 minutes or more of moderate or vigorous physical activity at least once per month vs no activity); monthly household income (Korean won, 1 USD = 1170 Korean won as of August 1, 2004; <500,000, 500,000–990,000, 1,000,000–1,490,000, ≥1,500,000, or missing information [n = 4798]). Mediators of the physiologic effects of BMI such as hypertension, diabetes, and dyslipidemia were not adjusted to show the full effects of BMI on mortality.^7^ Prevalent cancers and other diseases at baseline were not excluded to minimize the possibility of collider stratification bias and to facilitate generalizability to the general population.^20^ However, to investigate the potential influence of preexisting cancers, analyses were also performed in which individuals with preexisting cancers (n = 3465) and those with <2 years of follow-up (n = 2064, among whom 498 had preexisting cancer) were excluded. BMI was further classified into the standard 4 categories for between-study comparisons.^21^ The effect modification between BMI and smoking status (current vs never-smokers, past vs never-smokers) was assessed by including linear interaction terms in the Cox model. Subgroup analysis and analysis with different BMI categories served as sensitivity analyses. *P* values were calculated using 2-sided tests. All statistical analyses were performed using SAS version 9.4 (SAS Inc, Cary, NC).

### Ethics Approval

This study was approved by the Institutional Review Board of Kwandong University (Gangneung, Republic of Korea).

## RESULTS

During the median 6.4 years of follow-up (705,175 person-years), 3478 participants died from cancer. At baseline, the mean (standard deviation; range) age and BMI were 58.9 (3.5; 49.7–82.4) years and 23.6 (2.7; 12.3–46.9) kg/m^2^. The prevalence of underweight and obesity were 2.6% and 1.2%, respectively. The more obese men tended to be somewhat younger and were less likely to be current smokers (Table 1).

**TABLE 1:**
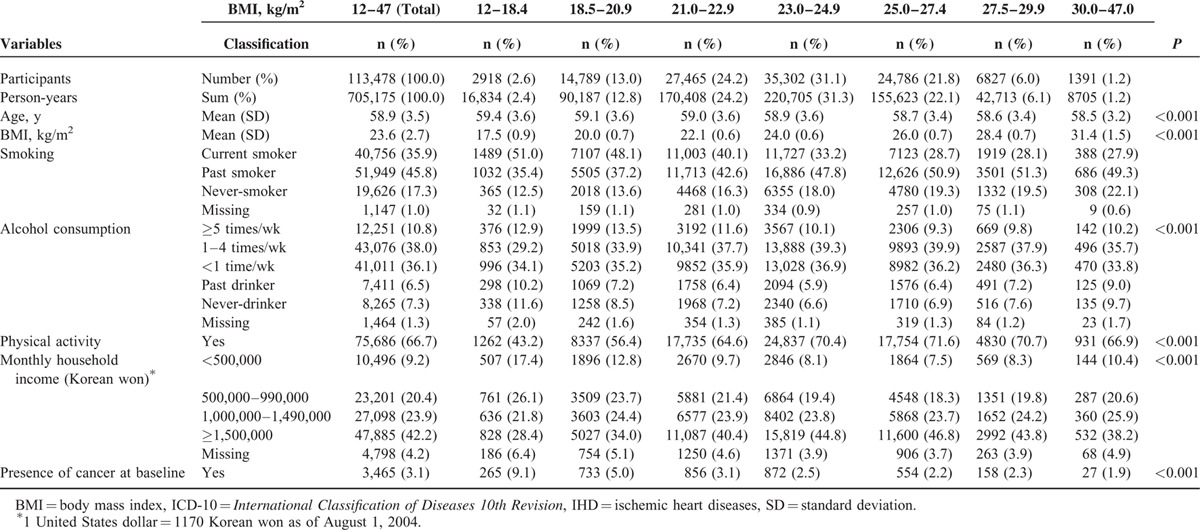
Baseline Characteristics and Follow-Up Duration by BMI Categories

**FIGURE 1:**
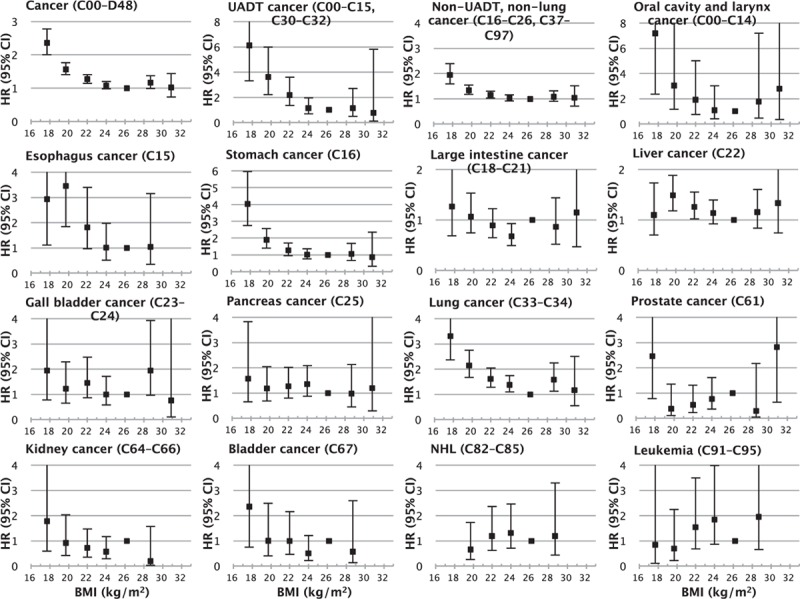
Hazard ratios for cancer mortality across 7 BMI categories. The midpoint BMI was used as a representative value for each category (kg/m^2^: 12–18.4, 18.5–20.9, 21–22.9, 23–24.9, 25–27.4 [reference], 27.5–29.9, 30.0–47), except for the highest and lowest BMI categories, in which the median was used. Analyses were adjusted for age, smoking, alcohol intake, household income, and physical activity. For some causes, no deaths were observed in the highest or the lowest BMI categories. BMI = body mass index, CI = confidence interval, HR = hazard ratio.

Both low BMI and high BMI were associated with higher mortality from all cancers combined (henceforth “all-cancer”) with reverse-J curve associations. Mortality from all-cancer was the lowest at 25–27.4 kg/m^2^ (Figure 1). Below 25 kg/m^2^, cancer mortality increased >70% for each 5 kg/m^2^ lower BMI (HR = 1.72, 95% confidence interval [CI] = 1.57–1.90), whereas in the range of 25–47 kg/m^2^, cancer mortality increased approximately 30% for each 5 kg/m^2^ higher BMI (Table 2, eFigure 1). Strong inverse associations for upper aerodigestive tract (UADT) cancers combined (henceforth “UADT cancer”) including oral cavity, larynx and esophagus cancers, and lung cancer were found, and non-UADT and non-lung cancers combined (henceforth “non-UADT-non-lung cancer”) including stomach and large intestine cancers were also inversely related to BMI. In the range of 25–47 kg/m^2^, the positive associations were mainly attributable to non-UADT-non-lung cancer (especially liver cancer and leukemia).

**TABLE 2:**
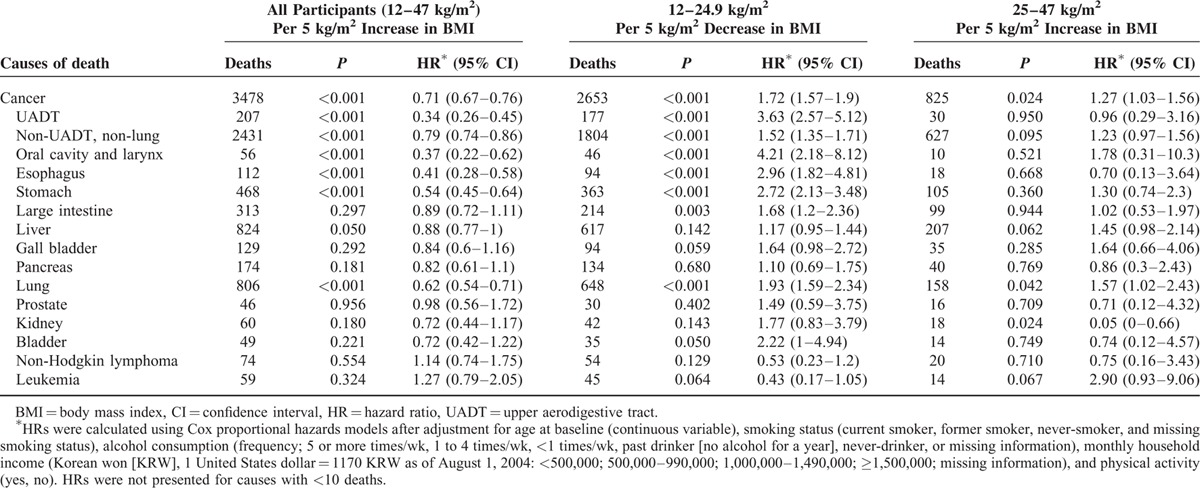
Cause-Specific Mortality Associated With Baseline BMI According to BMI Ranges

**FIGURE 2:**
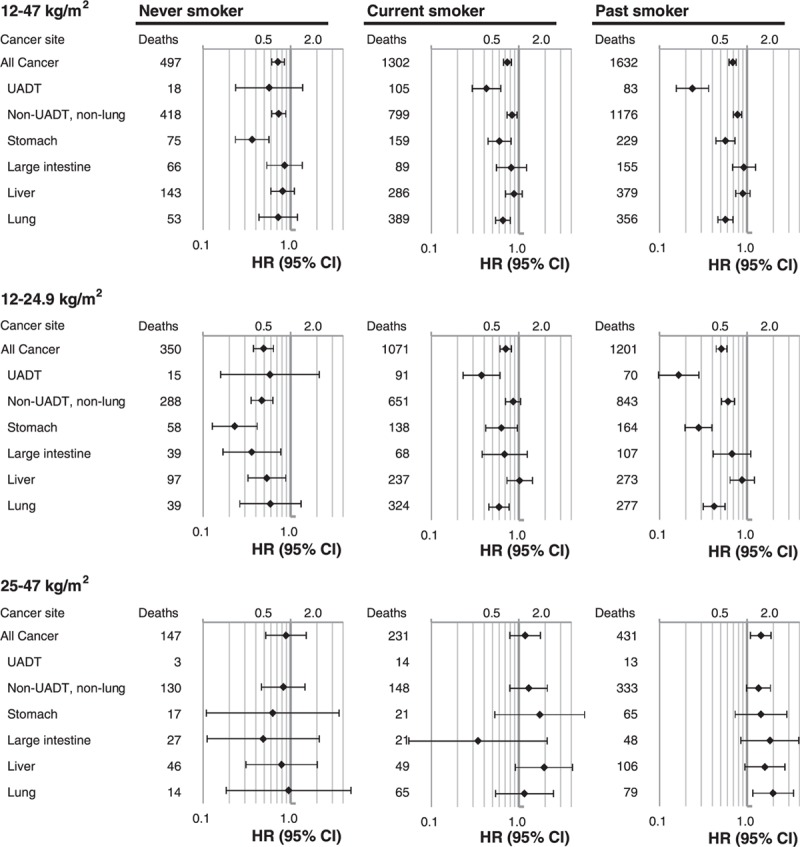
Hazard ratios for mortality from selected cancers per 5 kg/m^2^ increase in BMI across BMI groups stratified by smoking status. Analyses were adjusted for age, smoking, alcohol intake, household income, and physical activity. Cancers that had 10 or more cases in never-smokers in the 12–24.9 or 25–47 kg/m^2^ range were included. BMI = body mass index, CI = confidence interval, HR = hazard ratio.

In stratified analyses by smoking status (Figure 2), never-smokers had stronger inverse associations with BMI than current smokers in the range <25 kg/m^2^ for all-cancer mortality [*P*_interaction_ (current vs never-smokers) = 0.001] and non-UADT-non-lung cancer mortality (*P*_interaction_ < 0.001), whereas they had similar inverse associations for mortality from UADT cancer (*P*_interaction_ = 0.738) and lung cancer (*P*_interaction_ = 0.741). In never-smokers, in the range of 25 to 47 kg/m^2^, no positive linear association of BMI was found with mortality from all-cancer and from the major cancers analyzed. Ever-smokers (especially past smokers) had generally stronger positive associations than never-smokers, although the *P*-values for these interactions were >0.05. For example, *P*_interaction_ (past vs never-smokers) was 0.132 and 0.162 for mortality from all-cancer and non-UADT-non-lung cancer, respectively.

**FIGURE 3:**
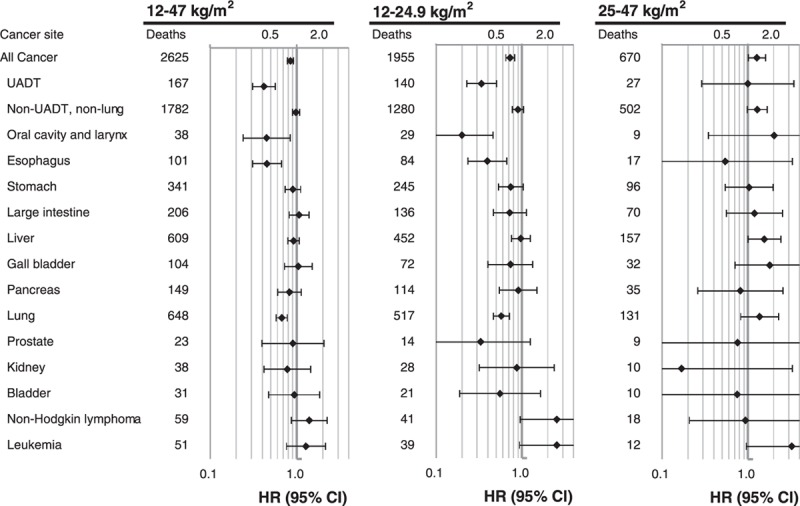
Hazard ratios for mortality from selected cancers per 5 kg/m^2^ increase in BMI across BMI groups in participants with no preexisting cancers. Analyses were adjusted for age, smoking, alcohol intake, household income, and physical activity. Cancers that had 10 or more cases in the 12–24.9 or 25–47 kg/m^2^ range among all participants were included. BMI = body mass index, CI = confidence interval, HR = hazard ratio.

After exclusion of preexisting cancers, and the first 2 years of follow-up, in the range <25 kg/m^2^, HRs of the inverse associations of BMI with mortality from all cancer and site-specific cancers were generally weakened, but remained significant with some exceptions. The *P*-values increased >0.05 for non-UADT-non-lung cancer, including the stomach, large intestine, and bladder cancer (Figure 3, eFigures. 2–4 eTable 2). In the range <25 kg/m^2^, after such exclusion, each 5 kg/m^2^ increase in BMI was associated with higher mortality from leukemia (HR = 3.25, 95% CI = 1.04–10.19) and non-Hodgkin lymphoma (HR = 3.59, 95% CI = 1.11–11.58). In the range of 25 to 47 kg/m^2^, after such exclusion, the magnitude of the positive associations of BMI with all cancer, non-UADT and non-lung cancer, and liver cancer were not weakened.

In the analyses using standard BMI categories, inverse associations of BMI with cancer mortality were generally observed. However, a U-curve association with BMI was found for mortality from prostate cancer (eTable 3).

## DISCUSSION

Among 113,478 Korean men, all-cancer mortality was the lowest at 25.0 to 27.4 kg/m^2^, after adjusting for smoking, drinking, household income, and physical activity. Reverse J-curve associations were observed for all-cancer mortality, whereas inverse associations for UADT cancer and lung cancer mortality were observed. In the range of 25 to 47 kg/m^2^, each 5 kg/m^2^ higher BMI was associated with approximately 30% higher all-cancer mortality, which was mainly attributable to non-UADT-non-lung cancer. Below 25 kg/m^2^, each 5 kg/m^2^ lower BMI was associated with approximately 70% higher all-cancer mortality (260% for UADT cancer; 50% for non-UADT-non-lung cancer [170% for stomach cancer; 70% for colorectal cancer]; and 90% for lung cancer). After excluding participants with preexisting cancers and the first 2 years of follow-up, the HRs associated with lower BMI was weakened (approximately 30% higher all-cancer mortality per each 5 kg/m^2^ lower BMI), especially for non-UADT and non-lung cancer. When stratified by smoking status, in the range <25 kg/m^2^, inverse associations of BMI with mortality from all cancer and non-UADT-non-lung cancer were stronger in never-smokers than in current smokers.

### Stomach Cancer

The strong inverse associations between BMI and stomach cancer mortality in the current study were similar to findings in people with stomach cancer.^22,23^ Individuals with a low BMI who are diagnosed with stomach cancer may not easily tolerate treatment-related stress and fasting.^23^ The association of low BMI with stomach cancer mortality was substantially weakened after exclusion of the early follow-up period and preexisting cancers. A low BMI may reflect poor nutritional status due to impaired oral intake resulting from more advanced stages of cancer.^22^ Thus, a low BMI may be a prognostic factor, or reflect reverse causation, rather than being a causal factor for stomach cancer. The findings that BMI >25 kg/m^2^ was not associated with higher stomach cancer mortality corresponded to those of other prospective studies on the incidence of stomach cancer.^2,24^

### Large Intestine Cancer

Among all participants, colorectal cancer mortality was associated with lower BMI, in accordance with the findings of a systematic review among colorectal cancer patients.^25^ However, in the analysis using standard BMI categories after the exclusion of the early follow-up period and preexisting cancers, this association was mitigated, whereas an association with higher BMI was found; overweight (HR = 1.30, 95% CI = 0.95–1.77) and obesity (HR = 2.48, 95% CI = 1.01–6.08) were associated with higher mortality than the normal weight. Moreover, each 5 kg/m^2^ increment in BMI was associated with higher mortality (HR = 1.61, 95% CI = 1.01–2.57) when the analysis was restricted to participants whose BMI was 23.0 kg/m^2^ (the cutoff point for overweight for Asians) or above. Lower BMI may be a reflection of cancer severity, other comorbidities, or the incapacity to survive cancer treatment.^25^ A higher incidence of colorectal cancer associated with higher BMI has been reported in previous research.^2,3^ These findings suggest a potential causal relationship between high BMI and colorectal cancer.

### Liver Cancer

Our study found evidence that may link liver cancer mortality with higher BMI in the range of 25 to 47 kg/m^2^. Liver cancer has been associated with higher BMI in many, but not all,^6^ previous studies.^2,3,7,26^ Although hepatitis is common in Korea, our study additionally adjusted for the presence of viral hepatitis and liver disease in participants.^26^ In the analysis including 7 BMI categories, participants with a BMI of 18.5 to 20.9 and 21.0 to 22.9 had a higher liver cancer mortality rate than those with a BMI of 25 to 27.4. Underweight was associated with liver cancer mortality in a previous study among Asians.^6^ Large prospective studies found associations between low BMI and liver diseases such as liver cirrhosis,^7,27^ which might partly explain the findings because cirrhosis is a risk factor for liver cancer. Overall, however, potential mechanism is not clear. More study is needed to confirm the association between BMI and liver cancer.

### UADT and Lung Cancer

Below 25 kg/m^2^, strong inverse associations of BMI with UADT (including esophagus, oral cavity, and larynx) and lung cancers were observed. Although no histologic information for esophageal cancer was available in the death records, the Korea Central Cancer Registry has reported that esophageal cancer in Koreans was generally squamous cell carcinoma.^5^ The inverse associations we observed concur with some, but not all,^28^ previous research.^2,5,29^ After the exclusion of the early follow-up period and preexisting cancers, these elevated risks were sustained in current and past smokers. In ever-smokers, the present study and previous research have found strong negative associations between UADT cancer and BMI even after exclusion of the early follow-up period, as well as a persistent inverse association after fine adjustment for smoking-related variables or a BMI calculation based on body weight several years before the cancer diagnosis, which suggests that explanations other than reverse causality and residual confounding by smoking should be considered.^7,30–32^

### Other Cancers

Kidney cancer was not associated with BMI, in contrast to some,^5,33^ but not all,^4^ previous studies among Asians. In the analysis using standard BMI categories, prostate cancer mortality was higher in the obese (≥30 kg/m^2^) group than in the normal weight group. In the current study, <25 kg/m^2^, each 5 kg/m^2^ increase in BMI was associated with higher mortality from leukemia and non-Hodgkin lymphoma after exclusion of preexisting cancers and the early follow-up period. With these restrictions, in the range of 12 to 47 kg/m^2^, non-Hodgkin lymphoma was associated with higher BMI (HR per 5 kg/m^2^ higher BMI = 1.68, 95% CI = 1.01–2.81). This association of non-Hodgkin lymphoma and leukemia is somewhat similar the results from previous studies in Asian populations.^4,5^ More research is needed to confirm the associations of BMI with these cancers in Asian populations.

### All-Cancer Mortality and Effect Modification by Smoking

We found that in the range <25 kg/m^2^, lower BMI was associated with higher all-cancer mortality, while in the range of 25 kg/m^2^ or above, higher BMI was associated with higher all-cancer mortality. This generally corresponds to previous studies in which there is a sufficient number of participants with low-normal weight and underweight.^4,7,28,34,35^ Mechanisms that may link adiposity, commonly assessed as BMI, to sex- and site-specific cancers have been suggested,^36,37^ but a clear understanding of how carcinogenesis or the prognosis of sex- and site-specific cancers may be associated with adiposity—both thinness and fatness—requires further research.

The inverse association with all-cancer mortality in the range <25 kg/m^2^ has been suggested to be primarily due to inverse associations with UADT and lung cancers in ever-smokers, whereas the positive association of higher BMI with all-cancer mortality has been suggested to be more profound in never-smokers than in ever-smokers.^7,12^ However, our findings did not support those suggestions. In the current study, in the range <25 kg/m^2^, the inverse association of BMI with all-cancer mortality was stronger in never-smokers (mainly due to strong inverse associations with non-UADT-non-lung cancer) than in current smokers, whereas the association of higher BMI with all-cancer mortality was weaker instead of stronger in never-smokers than in current or past smokers in the range 25 to 47 kg/m^2^. In a large Asian collaborative study, the association of lower BMI with lung cancer mortality was found to be stronger in never-smokers.^4^ The effect modification by smoking, if it exists, may vary across populations due to ethnic, regional, or other factors.^4,9^

### Implications

The associations between higher BMI and mortality from all cancers and several site-specific cancers could be causal. Regarding the BMI range <25 kg/m^2^, some evidence has suggested causality in the association between lower BMI and mortality and incidence from UADT and lung cancers.^7,30–32^ For stomach and colorectal cancers, our findings suggested that low BMI was instead a prognostic factor or reflected reverse causation. Regardless of causality, however, weight loss, if occurs, could have been taken place over years, if not decades, before the diagnosis of the cancers inversely associated with BMI. Because it is difficult to distinguish between intentional versus unintentional weight loss, maintaining a low-normal weight with advancing age may not be necessarily a sign of good health.^9,38^ Careful evaluation might increase the chance of diagnosing these cancers at an earlier stage in men with low-normal weight. Further evaluation is necessary to determine whether interventions involving intentional weight change (gain or loss) reduce the risk of cancer or improve the survival rate.

### Strengths and Limitations of this Study

The prospective design and complete follow-up using national mortality data are strengths of our study. Other strengths are that our results were drawn from a recent cohort,^34,39,40^ and that the number of deaths from cancers was one of the largest in studies among East Asian men.^4^ Additionally, preexisting cancers were identified using the National Cancer Incidence Database, meaning that bias related to the self-reporting of preexisting illnesses was minimized, and some conditions important to specific cancers were adjusted for in the analyses (e.g., viral hepatitis infection for liver cancer).

Our study also has several limitations. BMI was calculated based on self-reported height and weight.^41^ Data on self-reported smoking and other covariates could have led to residual confounding. The validity of the diagnosis on death certificates was not examined separately. However, Korean death certificate was reported to have reasonable validity compared with medical records.^42^ Additionally, potential misclassification of cause of death was considered generally nondifferential according to BMI. Since our study participants were relatively thin overall, our study had a limited ability to identify associations of the highest BMIs (e.g., 40 kg/m^2^ or above) with mortality. Due to a short follow-up period, the number of deaths was small for some less common cancers, especially in the analyses according to smoking status. However, a longer follow-up can also introduce bias due to considerable changes in body weight and other covariates over time, as well as the diagnosis and treatment of specific diseases.^34,39^

The inclusion of only Korean Vietnam War veterans in this study limits its generalizability. The study participants (mean age: 58.9) had a similar cancer mortality (493 per 100,000, [95% CI = 477–510]) to the nonparticipants (mean age: 58.4) who did not return the survey (504 per 100,000, [478–530]). Although our participants had military experience, as all able-bodied Korean men have mandatory military service, military experience *per se* may not reduce the generalizability of our findings to Korean men. Our participants had a lower mortality (standardized mortality ratio = 0.80, 95% CI = 0.79–0.82) than the general male population of Korea. However, we do not think that the lower mortality is likely to substantially change the observed associations between BMI and cancer mortality. Meanwhile, the longevity of the Korean population is comparable to that of the populations of other Organization for Economic Cooperation and Development countries (OECD).^43^ This aspect may make our main findings more generalizable to the OECD populations with similar longevity. However, the association of BMI with mortality may differ by sex, ethnicity, and region. Thus, some of our findings may not be applicable to other populations, especially those who are more obese, and women.

In conclusion, both low BMI and high BMI were found to be strong predictors of mortality from cancer overall and from several site-specific cancers in Korean men. A low BMI may increase the risk of UADT and lung cancers, especially in ever-smokers. Differences in the associations of BMI with mortality from various cancers suggest different biological mechanisms related to adiposity. Interventions involving weight change should be studied in order to determine whether intentional weight change (gain or loss) reduces the risk of cancer or improves survival.

### Novelty and Impact

The association of both low and high BMI with overall and site-specific cancer mortality in Asians is not well understood. This large prospective cohort study in Korean men showed that strong inverse associations for UADT (oral cavity, larynx, and esophagus), lung, stomach, and colorectal cancers were found <25 kg/m^2^. Further analysis excluding preexisting cancers and the early follow-up period suggest that low BMI (or factors closely related to low BMI) might cause UADT and lung cancers, especially in smokers, whereas it may be a prognostic factor or reflect reverse causation in stomach and colorectal cancers. Evidence was also found suggesting that higher BMIs may be linked with mortality from liver, colorectal, and prostate cancers, as well as leukemia and non-Hodgkin lymphoma.

## Supplementary Material

Supplemental Digital Content
