# Supplementary material for: Body Mass Index and Cancer Mortality Among Korean Older Middle-Aged Men: A Prospective Cohort Study
Source: Medicine (Baltimore). 2016 May 27;95(21):e3684. doi: 10.1097/MD.0000000000003684 (PMC4902352; doi:10.1097/MD.0000000000003684)
Supplement: Supplemental Digital Content [file medi-95-e3684-s001.pdf]

eTable 1. Cancer mortality outcomes, their ICD-10 codes, and characteristics related to exclusion of prevalent diseases at baseline (and two years of follow-up)

Cancer site	ICD-10	No. Deaths in all participants (n=113,478) (PY=705,175)	No. Deaths in participants with no pre-exiting cancer (n=110,013) (PY=686,845)	No. Deaths in participants with no pre-exiting cancer after exclusion of the first two years of follow-up (n=108,447) (PY=685,119)	Variables additionally adjusted for in the Cox model
All cancer	C00-D48	3478	2625	2164	
Upper aerodigestive tract (UADT)	C00-C15, C30-C32	207	167	139	
Non-UADT-non-lung	C16-C26, C37-C97	2431	1782	1462	
Oral cavity and larynx	C00-C14	56	38	32	
Esophagus	C15	112	101	85	
Stomach	C16	468	341	290	
Large intestine	C18-C21	313	206	191	
Liver	C22	824	609	469	Viral hepatitis (B15-B19) and liver diseases
Gall bladder	C23-C24	129	104	78	
Pancreas	C25	174	149	114	
Lung	C33-C34	806	648	543	Respiratory related diseases (COPD, pneumonia, tuberculosis)
Prostate	C61	46	23	22	
Kidney	C64-C66	60	38	32	
Bladder	C67	49	31	24	
Non-Hodgkin lymphoma (NHL)	C82-C85	74	59	49	
Leukemia	C91-C95	59	51	43	

BMI, body mass index; COPD, chronic obstructive pulmonary diseases; CVD, cerebrovascular diseases; HR, hazard ratio; ICD-10, International Classification of Diseases 10th Revision; IHD, ischemic heart disease; NHL, non-Hodgkin lymphoma; PY=person-year; SD, standard deviation; UADT, upper aero-digestive tract

eTable 2. Hazard ratios for cancer mortality associated with BMI across BMI groups in participants with no pre-existing cancers at baseline.

	All participants (12-47 kg/m²)				12-24.9 kg/m²				25-47 kg/m²			
		per 5 kg/m² **higher** BMI				per 5 kg/m² **lower** BMI				per 5 kg/m² **higher** BMI		
Cancer site	Deaths	p-value	HR[b]	(95% CI)	Deaths	p-value	HR[b]	(95% CI)	Deaths	p-value	HR[b]	(95% CI)
All cancer	2625	<0.001	0.85	(0.79, 0.91)	1955	<0.001	1.37	(1.22, 1.54)	670	0.039	1.27	(1.01, 1.61)
UADT	167	<0.001	0.42	(0.31, 0.57)	140	<0.001	2.95	(1.98, 4.39)	27	0.998	1.00	(0.29, 3.45)
Non-UADT, non-lung	1782	0.722	0.98	(0.9, 1.08)	1280	0.163	1.11	(0.96, 1.29)	502	0.064	1.29	(0.99, 1.68)
Oral cavity and larynx	38	0.012	0.45	(0.24, 0.84)	29	<0.001	4.99	(2.18, 11.42)	9	0.434	2.02	(0.35, 11.73)
Esophagus	101	<0.001	0.45	(0.31, 0.67)	84	0.001	2.53	(1.5, 4.27)	17	0.511	0.55	(0.09, 3.31)
Stomach	341	0.341	0.91	(0.74, 1.11)	245	0.076	1.35	(0.97, 1.88)	96	0.913	1.04	(0.54, 1.97)
Large intestine	206	0.624	1.07	(0.82, 1.39)	136	0.152	1.38	(0.89, 2.15)	70	0.650	1.19	(0.56, 2.52)
Liver	609	0.276	0.92	(0.79, 1.07)	452	0.813	1.03	(0.8, 1.33)	157	0.050	1.56	(1, 2.44)
Gall bladder	104	0.822	1.04	(0.72, 1.5)	72	0.314	1.36	(0.75, 2.49)	32	0.218	1.80	(0.71, 4.6)
Pancreas	149	0.231	0.82	(0.6, 1.13)	114	0.703	1.10	(0.66, 1.83)	35	0.725	0.81	(0.26, 2.55)
Lung	648	<0.001	0.67	(0.58, 0.78)	517	<0.001	1.74	(1.4, 2.16)	131	0.221	1.37	(0.83, 2.28)
Prostate	23	0.811	0.90	(0.4, 2.06)	14	0.103	3.02	(0.8, 11.41)	9	0.820	0.76	(0.07, 8.32)
Kidney	38	0.431	0.78	(0.42, 1.45)	28	0.784	1.15	(0.42, 3.14)	10	0.240	0.17	(0.01, 3.29)
Bladder	31	0.850	0.94	(0.48, 1.84)	21	0.291	1.80	(0.61, 5.33)	10	0.795	0.75	(0.09, 6.48)
Non-Hodgkin lymphoma	59	0.172	1.40	(0.87, 2.25)	41	0.063	0.39	(0.14, 1.05)	18	0.931	0.94	(0.21, 4.21)
Leukemia	51	0.352	1.28	(0.76, 2.15)	39	0.067	0.39	(0.14, 1.07)	12	0.059	3.24	(0.96, 10.96)

BMI, body mass index; CI, confidence interval; COPD, chronic obstructive pulmonary diseases; HR, hazard ratio; UADT, upper aero-digestive tract

[a] For excluded diseases and additionally adjusted diseases for each cause of death, see eTable1.

[b] Hazard ratios were calculated using Cox proportional hazards models after adjustment for age at baseline (continuous variable), smoking status (current smoker, former smoker, never-smoker, and those with missing smoking status), alcohol intake (frequency; 5 or more times/week, 1-4 times/week, less than 1 time/week, past drinkers [no alcohol for a year], never drinker, or those with missing information), monthly household income (Korean Won [KRW], 1 United States Dollar = 1170 KRW as of August 1, 2004; < 500,000, 500,000-990,000, 1,000,000-1,490,000, ≥ 1,500,000, those with missing information), and physical activity (yes, no). HRs were not presented for cancers with less than 5 deaths.

eTable 3. Hazard ratios for cancer mortality associated with standard four categories of BMI

		BMI	All participants					Participants with no pre-existing cancers at baseline[a]				
**Canser site**	**ICD-10**	**(kg/m²)**	**Deaths**	**Rate[b]**	**p-value**	**HR[c]**	**(95% CI)**	**Deaths**	**Rate[b]**	**p-value**	**HR[c]**	**(95% CI)**
All cancer	C00-D48	12-18.4	194	11,524	<0.001	1.87	(1.61, 2.17)	101	6,453	0.003	1.36	(1.11, 1.66)
		18.5-24.9	2459	5,109		1.00	(Reference)	1854	3,959		1.00	(Reference)
		25.0-29.9	789	3,978	<0.001	0.83	(0.77, 0.9)	642	3,303	0.081	0.92	(0.84, 1.01)
		30.0-47	36	4,136	0.257	0.83	(0.59, 1.15)	28	3,272	0.593	0.90	(0.62, 1.31)
UADT cancer	C00-C15,	12-18.4	20	1,188	<0.001	2.87	(1.79, 4.61)	13	831	0.003	2.38	(1.33, 4.24)
	C30-C32	18.5-24.9	157	326		1.00	(Reference)	127	271		1.00	(Reference)
		25.0-29.9	29	146	0.001	0.51	(0.34, 0.77)	26	134	0.013	0.58	(0.38, 0.89)
		30.0-47	1	115	0.343	0.39	(0.05, 2.76)	1	117	0.485	0.50	(0.07, 3.55)
Non-UADT,	C16-C26,	12-18.4	118	7,010	<0.001	1.71	(1.41, 2.06)	51	3,259	0.537	1.09	(0.82, 1.45)
non-lung cancer	C37-C97	18.5-24.9	1686	3,503		1.00	(Reference)	1229	2,624		1.00	(Reference)
		25.0-29.9	599	3,020	0.031	0.90	(0.82, 0.99)	480	2,470	0.866	1.01	(0.91, 1.12)
		30.0-47	28	3,217	0.623	0.91	(0.63, 1.32)	22	2,571	0.857	1.04	(0.68, 1.59)
Oral cavity and	C00-C14	12-18.4	7	416	0.001	3.84	(1.69, 8.71)	6	383	<0.001	6.14	(2.46, 15.38)
larynx cancer		18.5-24.9	39	81		1.00	(Reference)	23	49		1.00	(Reference)
		25.0-29.9	9	45	0.253	0.65	(0.32, 1.35)	8	41	0.981	1.01	(0.45, 2.27)
		30.0-47	1	115	0.670	1.54	(0.21, 11.23)	1	117	0.328	2.72	(0.37, 20.22)
Esophagus cancer	C15	12-18.4	6	356	0.302	1.55	(0.67, 3.58)	4	256	0.780	1.16	(0.42, 3.18)
		18.5-24.9	88	183		1.00	(Reference)	80	171		1.00	(Reference)
		25.0-29.9	18	91	0.032	0.57	(0.34, 0.95)	17	87	0.062	0.60	(0.36, 1.02)
		30.0-47	0	0		-		0	0		-	
Stomach cancer	C16	12-18.4	41	2,436	<0.001	3.09	(2.22, 4.3)	9	575	0.912	0.96	(0.49, 1.88)
		18.5-24.9	322	669		1.00	(Reference)	236	504		1.00	(Reference)
		25.0-29.9	101	509	0.045	0.79	(0.63, 1)	93	479	0.757	1.04	(0.82, 1.32)
		30.0-47	4	460	0.435	0.67	(0.25, 1.81)	3	351	0.622	0.75	(0.24, 2.35)
Large intestine cancer	C18-C21	12-18.4	12	713	0.164	1.52	(0.84, 2.73)	7	447	0.320	1.47	(0.69, 3.17)
		18.5-24.9	202	420		1.00	(Reference)	129	275		1.00	(Reference)
		25.0-29.9	94	474	0.192	1.18	(0.92, 1.51)	65	334	0.085	1.30	(0.96, 1.76)
		30.0-47	5	574	0.466	1.39	(0.57, 3.38)	5	584	0.067	2.31	(0.94, 5.64)
Liver cancer[d]	C22	12-18.4	22	1,307	0.537	0.87	(0.57, 1.34)	13	831	0.347	0.77	(0.44, 1.33)
		18.5-24.9	595	1,236		1.00	(Reference)	439	937		1.00	(Reference)
		25.0-29.9	195	983	0.025	0.83	(0.71, 0.98)	148	761	0.036	0.82	(0.68, 0.99)
		30.0-47	12	1,379	0.817	1.07	(0.6, 1.9)	9	1,052	0.789	1.09	(0.57, 2.12)
Gall bladder cancer	C23-C24	12-18.4	6	356	0.271	1.60	(0.69, 3.67)	5	319	0.185	1.86	(0.74, 4.64)
		18.5-24.9	88	183		1.00	(Reference)	67	143		1.00	(Reference)
		25.0-29.9	34	171	0.986	1.00	(0.67, 1.5)	31	160	0.375	1.21	(0.79, 1.87)
		30.0-47	1	115	0.644	0.63	(0.09, 4.51)	1	117	0.883	0.86	(0.12, 6.22)
Pancreas cancer	C25	12-18.4	6	356	0.625	1.23	(0.54, 2.8)	3	192	0.650	0.77	(0.24, 2.42)
		18.5-24.9	128	266		1.00	(Reference)	111	237		1.00	(Reference)
		25.0-29.9	38	192	0.162	0.77	(0.54, 1.11)	33	170	0.192	0.77	(0.52, 1.14)
		30.0-47	2	230	0.919	0.93	(0.23, 3.76)	2	234	0.914	1.08	(0.27, 4.38)
Lung cancer[e]	C33-C34	12-18.4	54	3,208	<0.001	2.01	(1.51, 2.66)	35	2,236	0.011	1.57	(1.11, 2.22)
		18.5-24.9	594	1,234		1.00	(Reference)	482	1,029		1.00	(Reference)
		25.0-29.9	151	761	<0.001	0.70	(0.58, 0.83)	126	648	0.003	0.74	(0.61, 0.91)
		30.0-47	7	804	0.387	0.72	(0.34, 1.52)	5	584	0.374	0.67	(0.28, 1.62)
Prostate cancer	C61	12-18.4	4	238	0.010	4.08	(1.4, 11.92)	2	128	0.021	6.02	(1.31, 27.7)
		18.5-24.9	26	54		1.00	(Reference)	12	26		1.00	(Reference)
		25.0-29.9	14	71	0.344	1.37	(0.71, 2.64)	8	41	0.284	1.64	(0.66, 4.03)
		30.0-47	2	230	0.038	4.61	(1.09, 19.5)	1	117	0.118	5.12	(0.66, 39.68)
Kidney cancer	C64-C66	12-18.4	4	238	0.078	2.55	(0.9, 7.24)	1	64	0.974	1.03	(0.14, 7.69)
		18.5-24.9	38	79		1.00	(Reference)	27	58		1.00	(Reference)
		25.0-29.9	18	91	0.536	1.20	(0.68, 2.1)	10	51	0.859	0.94	(0.45, 1.94)
		30.0-47	0	0		-		0	0		-	
Bladder cancer	C67	12-18.4	4	238	0.042	2.99	(1.04, 8.6)	1	64	0.841	1.23	(0.16, 9.26)
		18.5-24.9	31	64		1.00	(Reference)	20	43		1.00	(Reference)
		25.0-29.9	14	71	0.624	1.17	(0.62, 2.21)	10	51	0.426	1.36	(0.63, 2.93)
		30.0-47	0	0		-		0	0		-	
NHL	C82-C85	12-18.4	0	0		-		0	0		-	
		18.5-24.9	54	112		1.00	(Reference)	41	88		1.00	(Reference)
		25.0-29.9	20	101	0.697	0.90	(0.54, 1.51)	18	93	0.770	1.09	(0.62, 1.9)
		30.0-47	0	0		-		0	0		-	
Leukemia	C91-C95	12-18.4	1	59	0.580	0.57	(0.08, 4.16)	0	0		-	
		18.5-24.9	44	91		1.00	(Reference)	39	83		1.00	(Reference)
		25.0-29.9	14	71	0.463	0.80	(0.44, 1.46)	12	62	0.379	0.75	(0.39, 1.43)
		30.0-47	0	0		-		0	0		-	

BMI, body mass index; CI, confidence interval; COPD, chronic obstructive pulmonary diseases; CVD, cerebrovascular diseases; HR, hazard ratio; ICD-10, International Classification of Diseases 10th Revision; IHD, ischemic heart disease; NHL, non-Hodgkin lymphoma; UADT, upper aero-digestive tract

[a] For pre-existing diseases excluded for each cause of death, see eTable 1.

[b] Crude death rate per 1,000,000 person-years

[c] Hazard ratios were calculated using Cox proportional hazards models after adjustment for age at baseline (continuous variable), smoking status (current smoker, former smoker, never-smoker, and those with missing smoking status), alcohol intake (frequency; 5 or more times/week, 1-4 times/week, less than 1 time/week, past drinkers [no alcohol for a year], never drinker, or those with missing information), monthly household income (Korean Won [KRW], 1 United States Dollar = 1170 KRW as of August 1, 2004; < 500,000, 500,000-990,000, 1,000,000-1,490,000, ≥ 1,500,000, those with missing information), and physical activity (yes, no).

[d] Additional adjustment for viral hepatitis and liver diseases at baseline was done in the Cox model.

[e] Additional adjustment for respiratory diseases (COPD, pneumonia, and tuberculosis) at baseline was done in the Cox model.

eTable 4. Hazard ratios for cancer mortality associated with seven categories of BMI (Numerical version of Figures 1, and eFigures 2)

		BMI	All participants					Participants with no pre-existing cancers at baseline[a]				
Cancer site	ICD-10	(kg/m^2)	Deaths	Rate[b]	p-value	HR[c]	(95% CI)	Deaths	Rate[b]	p-value	HR[c]	(95% CI)
All cancer	C00-D48	12-18.4	194	11,524	<0.001	2.36	(2, 2.78)	101	6453	<0.001	1.54	(1.24, 1.91)
		18.5-20.9	623	6,908	<0.001	1.57	(1.4, 1.76)	419	4848	0.001	1.26	(1.11, 1.44)
		21.0-22.9	893	5,240	<0.001	1.27	(1.14, 1.41)	696	4197	0.006	1.18	(1.05, 1.32)
		23.0-24.9	943	4,273	0.126	1.08	(0.98, 1.2)	739	3421	0.752	1.02	(0.91, 1.14)
		25.0-27.4	596	3,830		1.00	(Reference)	487	3193		1.00	(Reference)
		27.5-29.9	193	4,519	0.067	1.16	(0.99, 1.37)	155	3705	0.110	1.16	(0.97, 1.39)
		30.0-47	36	4,136	0.864	1.03	(0.74, 1.44)	28	3272	0.943	1.01	(0.69, 1.48)
UADT cancer	C00-C15,	12-18.4	20	1,188	<0.001	6.13	(3.3, 11.38)	13	831	<0.001	4.38	(2.15, 8.93)
	C30-C32	18.5-20.9	58	643	<0.001	3.63	(2.21, 5.98)	43	498	<0.001	2.87	(1.67, 4.92)
		21.0-22.9	61	358	0.002	2.20	(1.35, 3.6)	50	302	0.014	1.93	(1.14, 3.25)
		23.0-24.9	38	172	0.593	1.15	(0.68, 1.95)	34	157	0.697	1.12	(0.64, 1.94)
		25.0-27.4	22	141		1.00	(Reference)	20	131		1.00	(Reference)
		27.5-29.9	7	164	0.743	1.15	(0.49, 2.7)	6	143	0.843	1.10	(0.44, 2.73)
		30.0-47	1	115	0.813	0.79	(0.11, 5.83)	1	117	0.897	0.88	(0.12, 6.53)
Non-UADT,	C16-C26,	12-18.4	118	7,010	<0.001	1.95	(1.59, 2.39)	51	3259	0.477	1.11	(0.83, 1.5)
non-lung cancer	C37-C97	18.5-20.9	395	4,380	<0.001	1.34	(1.17, 1.54)	237	2742	0.989	1.00	(0.85, 1.18)
		21.0-22.9	611	3,586	0.021	1.15	(1.02, 1.3)	468	2822	0.248	1.08	(0.95, 1.24)
		23.0-24.9	680	3,081	0.706	1.02	(0.91, 1.15)	524	2426	0.663	0.97	(0.85, 1.11)
		25.0-27.4	460	2,956		1.00	(Reference)	367	2406		1.00	(Reference)
		27.5-29.9	139	3,254	0.408	1.08	(0.9, 1.31)	113	2701	0.291	1.12	(0.91, 1.38)
		30.0-47	28	3,217	0.879	1.03	(0.7, 1.51)	22	2571	0.800	1.06	(0.69, 1.63)
Oral cavity and	C00-C14	12-18.4	7	416	0.001	7.20	(2.36, 21.94)	6	383	0.001	7.83	(2.32, 26.5)
larynx cancer		18.5-20.9	14	155	0.024	3.04	(1.16, 8.02)	7	81	0.338	1.77	(0.55, 5.65)
		21.0-22.9	15	88	0.173	1.94	(0.75, 5.02)	10	60	0.462	1.50	(0.51, 4.41)
		23.0-24.9	10	45	0.852	1.10	(0.4, 3.03)	6	28	0.672	0.77	(0.24, 2.54)
		25.0-27.4	6	39		1.00	(Reference)	5	33		1.00	(Reference)
		27.5-29.9	3	70	0.407	1.80	(0.45, 7.19)	3	72	0.295	2.15	(0.51, 9)
		30.0-47	1	115	0.343	2.79	(0.33, 23.18)	1	117	0.266	3.38	(0.39, 29.02)
Esophagus cancer	C15	12-18.4	6	356	0.030	2.93	(1.11, 7.76)	4	256	0.250	1.94	(0.63, 5.99)
		18.5-20.9	35	388	<0.001	3.46	(1.84, 6.5)	30	347	0.001	2.90	(1.52, 5.53)
		21.0-22.9	32	188	0.066	1.81	(0.96, 3.4)	29	175	0.150	1.60	(0.84, 3.05)
		23.0-24.9	21	95	0.996	1.00	(0.51, 1.97)	21	97	0.974	0.99	(0.5, 1.95)
		25.0-27.4	14	90		1.00	(Reference)	14	92		1.00	(Reference)
		27.5-29.9	4	94	0.950	1.04	(0.34, 3.15)	3	72	0.702	0.78	(0.23, 2.73)
		30.0-47	0	0		-		0	0	0.985	0.00	(0, 0)
Stomach cancer	C16	12-18.4	41	2,436	<0.001	4.04	(2.75, 5.95)	9	575	0.796	0.91	(0.45, 1.83)
		18.5-20.9	94	1,042	<0.001	1.90	(1.4, 2.58)	59	683	0.355	1.18	(0.83, 1.67)
		21.0-22.9	114	669	0.097	1.28	(0.96, 1.71)	79	476	0.421	0.88	(0.64, 1.21)
		23.0-24.9	114	517	0.912	1.02	(0.76, 1.36)	98	454	0.418	0.88	(0.65, 1.19)
		25.0-27.4	78	501		1.00	(Reference)	75	492		1.00	(Reference)
		27.5-29.9	23	538	0.817	1.06	(0.66, 1.68)	18	430	0.610	0.87	(0.52, 1.46)
		30.0-47	4	460	0.776	0.86	(0.32, 2.36)	3	351	0.552	0.70	(0.22, 2.23)
Large intestine cancer	C18-C21	12-18.4	12	713	0.449	1.27	(0.69, 2.35)	7	447	0.814	1.10	(0.5, 2.44)
		18.5-20.9	50	554	0.719	1.07	(0.74, 1.54)	25	289	0.252	0.75	(0.47, 1.22)
		21.0-22.9	77	452	0.470	0.89	(0.65, 1.22)	55	332	0.540	0.89	(0.61, 1.3)
		23.0-24.9	75	340	0.017	0.68	(0.49, 0.93)	49	227	0.017	0.62	(0.42, 0.92)
		25.0-27.4	76	488		1.00	(Reference)	53	348		1.00	(Reference)
		27.5-29.9	18	421	0.569	0.86	(0.52, 1.44)	12	287	0.571	0.83	(0.45, 1.56)
		30.0-47	5	574	0.763	1.15	(0.46, 2.84)	5	584	0.252	1.71	(0.68, 4.28)
Liver cancer[d]	C22	12-18.4	22	1,307	0.673	1.10	(0.7, 1.73)	13	831	0.994	1.00	(0.56, 1.78)
		18.5-20.9	143	1,586	0.001	1.49	(1.18, 1.88)	92	1065	0.028	1.37	(1.03, 1.82)
		21.0-22.9	214	1,256	0.031	1.26	(1.02, 1.56)	162	977	0.016	1.35	(1.06, 1.73)
		23.0-24.9	238	1,078	0.231	1.13	(0.92, 1.39)	185	856	0.100	1.22	(0.96, 1.55)
		25.0-27.4	147	945		1.00	(Reference)	109	715		1.00	(Reference)
		27.5-29.9	48	1,124	0.371	1.16	(0.84, 1.61)	39	932	0.210	1.26	(0.88, 1.82)
		30.0-47	12	1,379	0.333	1.34	(0.74, 2.41)	9	1052	0.315	1.42	(0.72, 2.8)
Gall bladder cancer	C23-C24	12-18.4	6	356	0.156	1.94	(0.78, 4.84)	5	319	0.225	1.85	(0.68, 5)
		18.5-20.9	18	200	0.538	1.22	(0.65, 2.29)	8	93	0.213	0.59	(0.26, 1.35)
		21.0-22.9	38	223	0.160	1.46	(0.86, 2.48)	34	205	0.222	1.41	(0.81, 2.47)
		23.0-24.9	32	145	0.983	0.99	(0.58, 1.71)	25	116	0.576	0.85	(0.47, 1.52)
		25.0-27.4	22	141		1.00	(Reference)	20	131		1.00	(Reference)
		27.5-29.9	12	281	0.065	1.94	(0.96, 3.92)	11	263	0.070	1.98	(0.95, 4.12)
		30.0-47	1	115	0.785	0.76	(0.1, 5.62)	1	117	0.884	0.86	(0.12, 6.42)

Cancer site	ICD-10	BMI (kg/m^2)	All participants					Participants with no pre-existing cancers at baseline[a]				
			Deaths	Rate[b]	p-value	HR[c]	(95% CI)	Deaths	Rate[b]	p-value	HR[c]	(95% CI)
Pancreas cancer	C25	12-18.4	6	356	0.315	1.58	(0.65, 3.82)	3	192	0.935	0.95	(0.29, 3.16)
		18.5-20.9	23	255	0.550	1.18	(0.68, 2.05)	18	208	0.873	1.05	(0.57, 1.92)
		21.0-22.9	45	264	0.315	1.27	(0.8, 2.02)	44	265	0.189	1.38	(0.85, 2.24)
		23.0-24.9	60	272	0.181	1.35	(0.87, 2.09)	49	227	0.410	1.22	(0.76, 1.95)
		25.0-27.4	30	193		1.00	(Reference)	27	177		1.00	(Reference)
		27.5-29.9	8	187	0.948	0.97	(0.45, 2.13)	6	143	0.652	0.82	(0.34, 1.98)
		30.0-47	2	230	0.804	1.20	(0.29, 5.02)	2	234	0.686	1.34	(0.32, 5.66)
Lung cancer[e]	C33-C34	12-18.4	54	3,208	<0.001	3.31	(2.37, 4.61)	35	2236	<0.001	2.34	(1.57, 3.48)
		18.5-20.9	164	1,818	<0.001	2.14	(1.67, 2.75)	135	1562	<0.001	1.92	(1.47, 2.52)
		21.0-22.9	212	1,244	<0.001	1.62	(1.28, 2.04)	171	1031	0.005	1.44	(1.12, 1.86)
		23.0-24.9	218	988	0.006	1.38	(1.1, 1.75)	176	815	0.071	1.26	(0.98, 1.63)
		25.0-27.4	105	675		1.00	(Reference)	91	597		1.00	(Reference)
		27.5-29.9	46	1,077	0.009	1.58	(1.12, 2.24)	35	837	0.091	1.40	(0.95, 2.07)
		30.0-47	7	804	0.692	1.17	(0.54, 2.51)	5	584	0.969	0.98	(0.4, 2.42)
Prostate cancer	C61	12-18.4	4	238	0.125	2.46	(0.78, 7.79)	2	128	0.150	3.29	(0.65, 16.58)
		18.5-20.9	3	33	0.136	0.38	(0.11, 1.35)	1	12	0.227	0.27	(0.03, 2.25)
		21.0-22.9	8	47	0.172	0.54	(0.22, 1.31)	5	30	0.481	0.66	(0.21, 2.09)
		23.0-24.9	15	68	0.478	0.76	(0.36, 1.61)	6	28	0.318	0.57	(0.19, 1.71)
		25.0-27.4	13	84		1.00	(Reference)	7	46		1.00	(Reference)
		27.5-29.9	1	23	0.225	0.28	(0.04, 2.17)	1	24	0.576	0.55	(0.07, 4.47)
		30.0-47	2	230	0.173	2.82	(0.63, 12.57)	1	117	0.332	2.83	(0.35, 23.11)
Kidney cancer	C64-C66	12-18.4	4	238	0.304	1.79	(0.59, 5.43)	1	64	0.987	0.98	(0.12, 7.91)
		18.5-20.9	10	111	0.840	0.92	(0.42, 2.04)	6	69	0.848	1.11	(0.39, 3.16)
		21.0-22.9	14	82	0.363	0.72	(0.35, 1.46)	12	72	0.718	1.17	(0.49, 2.8)
		23.0-24.9	14	63	0.122	0.57	(0.28, 1.16)	9	42	0.422	0.68	(0.27, 1.73)
		25.0-27.4	17	109		1.00	(Reference)	9	59		1.00	(Reference)
		27.5-29.9	1	23	0.127	0.21	(0.03, 1.56)	1	24	0.393	0.41	(0.05, 3.21)
		30.0-47	0	0		-		0	0		-	
Bladder cancer	C67	12-18.4	4	238	0.145	2.37	(0.74, 7.53)	1	64	0.836	0.80	(0.1, 6.48)
		18.5-20.9	8	89	0.995	1.00	(0.4, 2.48)	5	58	0.692	0.80	(0.26, 2.43)
		21.0-22.9	14	82	0.983	0.99	(0.46, 2.15)	10	60	0.817	0.90	(0.36, 2.23)
		23.0-24.9	9	41	0.125	0.51	(0.21, 1.21)	5	23	0.069	0.36	(0.12, 1.08)
		25.0-27.4	12	77		1.00	(Reference)	9	59		1.00	(Reference)
		27.5-29.9	2	47	0.477	0.58	(0.13, 2.6)	1	24	0.377	0.39	(0.05, 3.11)
		30.0-47	0	0		-		0	0		-	
NHL	C82-C85	12-18.4	0	0		-		0	0		-	
		18.5-20.9	6	67	0.397	0.66	(0.25, 1.72)	3	35	0.127	0.37	(0.11, 1.32)
		21.0-22.9	20	117	0.594	1.20	(0.61, 2.35)	16	96	0.833	1.08	(0.52, 2.26)
		23.0-24.9	28	127	0.393	1.31	(0.7, 2.46)	22	102	0.643	1.18	(0.59, 2.34)
		25.0-27.4	15	96		1.00	(Reference)	13	85		1.00	(Reference)
		27.5-29.9	5	117	0.729	1.20	(0.43, 3.29)	5	120	0.538	1.38	(0.49, 3.88)
		30.0-47	0	0		-		0	0		-	
Leukemia	C91-C95	12-18.4	1	59	0.875	0.85	(0.11, 6.75)	0	0		-	
		18.5-20.9	4	44	0.533	0.69	(0.21, 2.24)	3	35	0.632	0.72	(0.18, 2.79)
		21.0-22.9	16	94	0.300	1.54	(0.68, 3.51)	16	96	0.113	2.06	(0.84, 5.02)
		23.0-24.9	24	109	0.116	1.85	(0.86, 3.98)	20	93	0.113	2.01	(0.85, 4.75)
		25.0-27.4	9	58		1.00	(Reference)	7	46		1.00	(Reference)
		27.5-29.9	5	117	0.230	1.95	(0.65, 5.83)	5	120	0.113	2.53	(0.8, 7.96)
		30.0-47	0	0		-		0	0		-	

BMI, body mass index; CI, confidence interval; COPD, chronic obstructive pulmonary diseases; CVD, cerebrovascular diseases; HR, hazard ratio; ICD-10, International Classification of Diseases 10th Revision; IHD, ischemic heart disease; NHL, non-Hodgkin lymphoma; UADT, upper aero-digestive tract

[a] For pre-existing diseases excluded for each cause of death, see eTable 1.

[b] Crude death rate per 1,000,000 person-years

[c] Hazard ratios were calculated using Cox proportional hazards models after adjustment for age at baseline (continuous variable), smoking status (current smoker, former smoker, never-smoker, and those with missing smoking status), alcohol intake (frequency; 5 or more times/week, 1-4 times/week, less than 1 time/week, past drinkers [no alcohol for a year], never drinker, or those with missing information), monthly household income (Korean Won [KRW], 1 United States Dollar = 1170 KRW as of August 1, 2004; < 500,000, 500,000-990,000, 1,000,000-1,490,000, ≥ 1,500,000, those with missing information), and physical activity (yes, no).

[d] Additional adjustment for viral hepatitis and liver diseases at baseline was done in the Cox model.

[e] Additional adjustment for respiratory diseases (COPD, pneumonia, and tuberculosis) at baseline was doen in the Cox model.

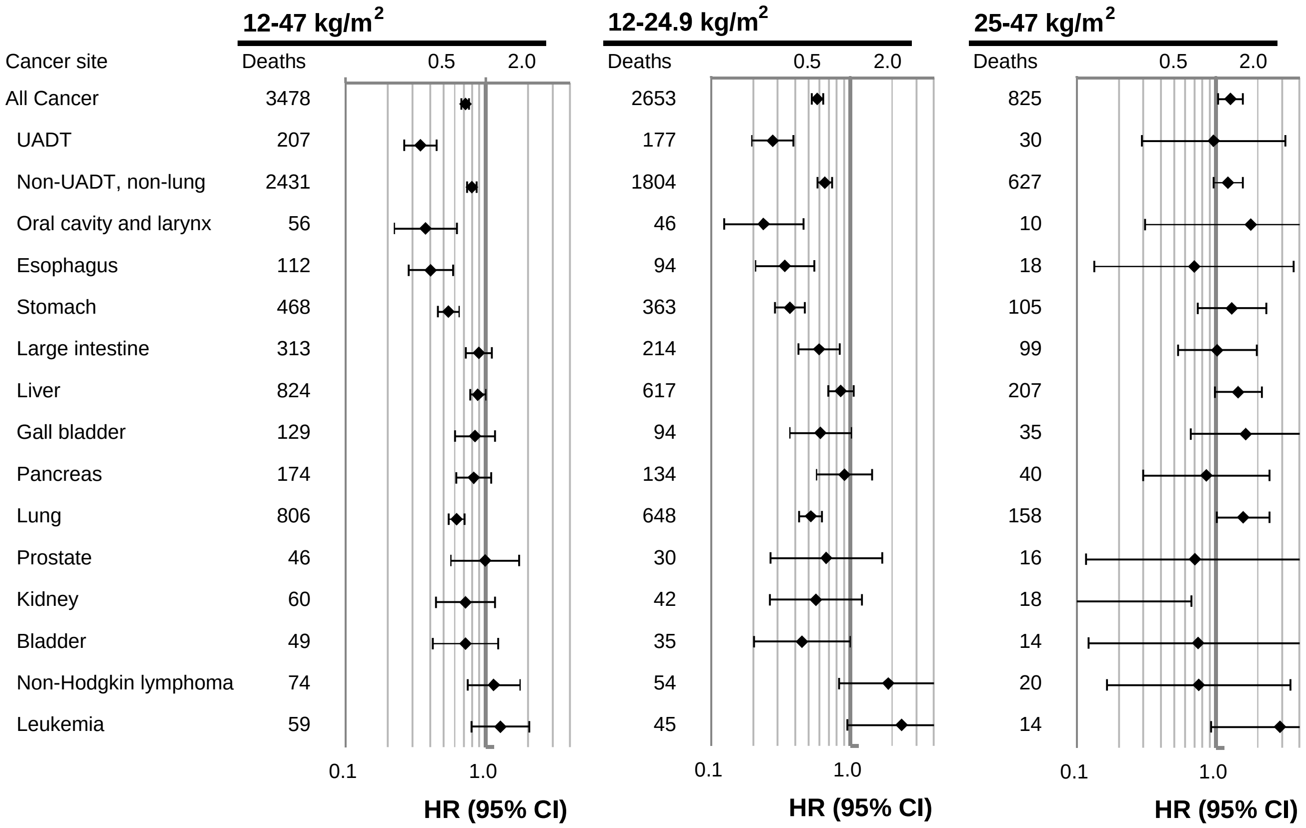

eFigure 1. Hazard ratios for cancer mortality per 5 kg/m^2 higher BMI across BMI groups. Analyses were adjusted for age, smoking, alcohol intake, household income, and physical activity. Cancers which had 10 or more cases in 12-24.9 or 25-47 kg/m^2 among all participants were included.

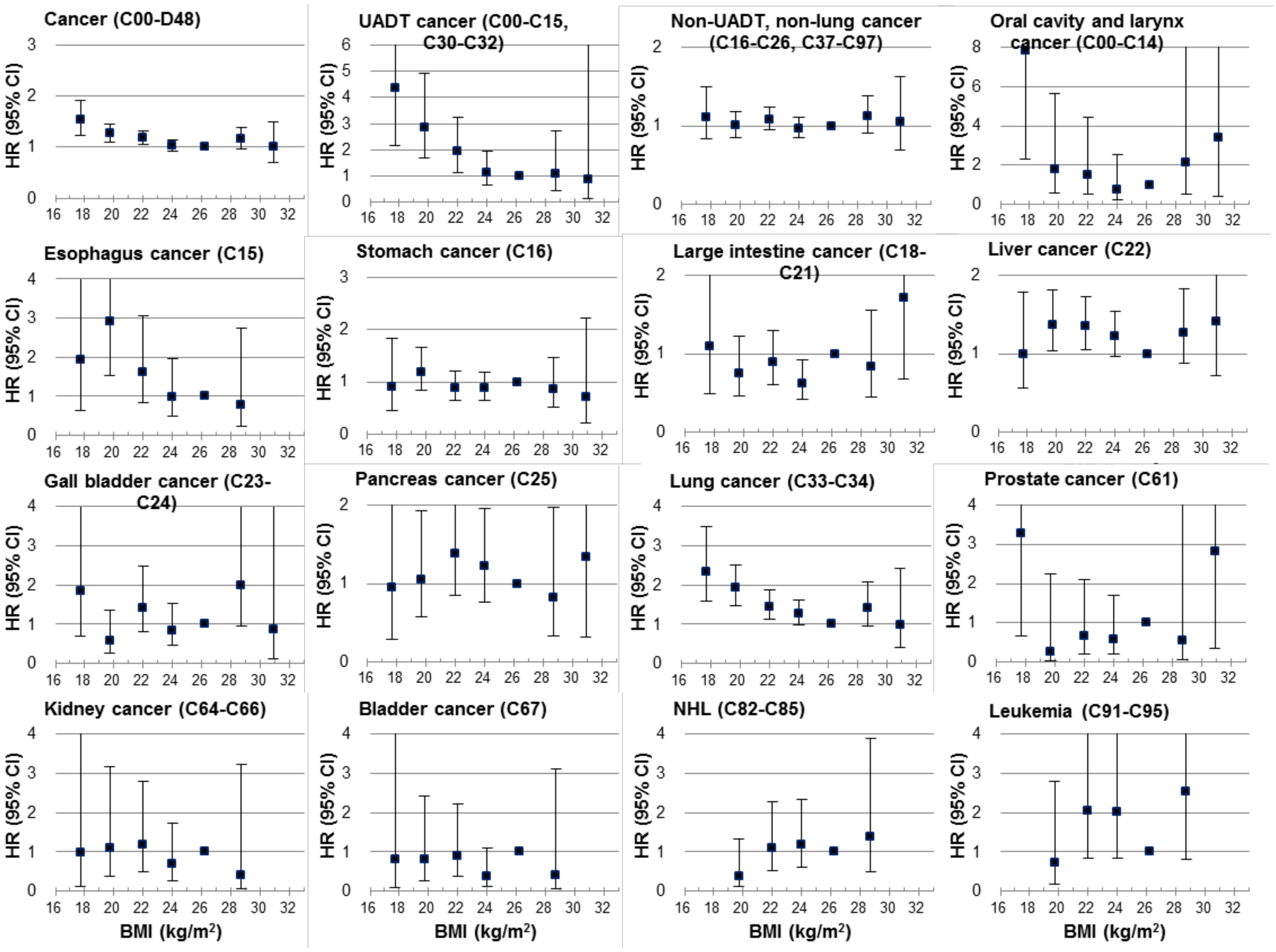

eFigure 2. Hazard ratios for cancer mortality in participants with no pre-existing cancers at baseline across seven categories of BMI. The midpoint BMI was used as a representative value for each category(kg/m^2; 12-18.4, 18.5-20.9, 21-22.9, 23-24.9, 25-27.4[Reference], 27.5-29.9, 30.0-47), except for both ends of BMI categories, in which the median was used. Analyses were adjusted for age, smoking, alcohol intake, household income, and physical activity. For some causes, no death was observed in the highest or the lowest BMI categories.

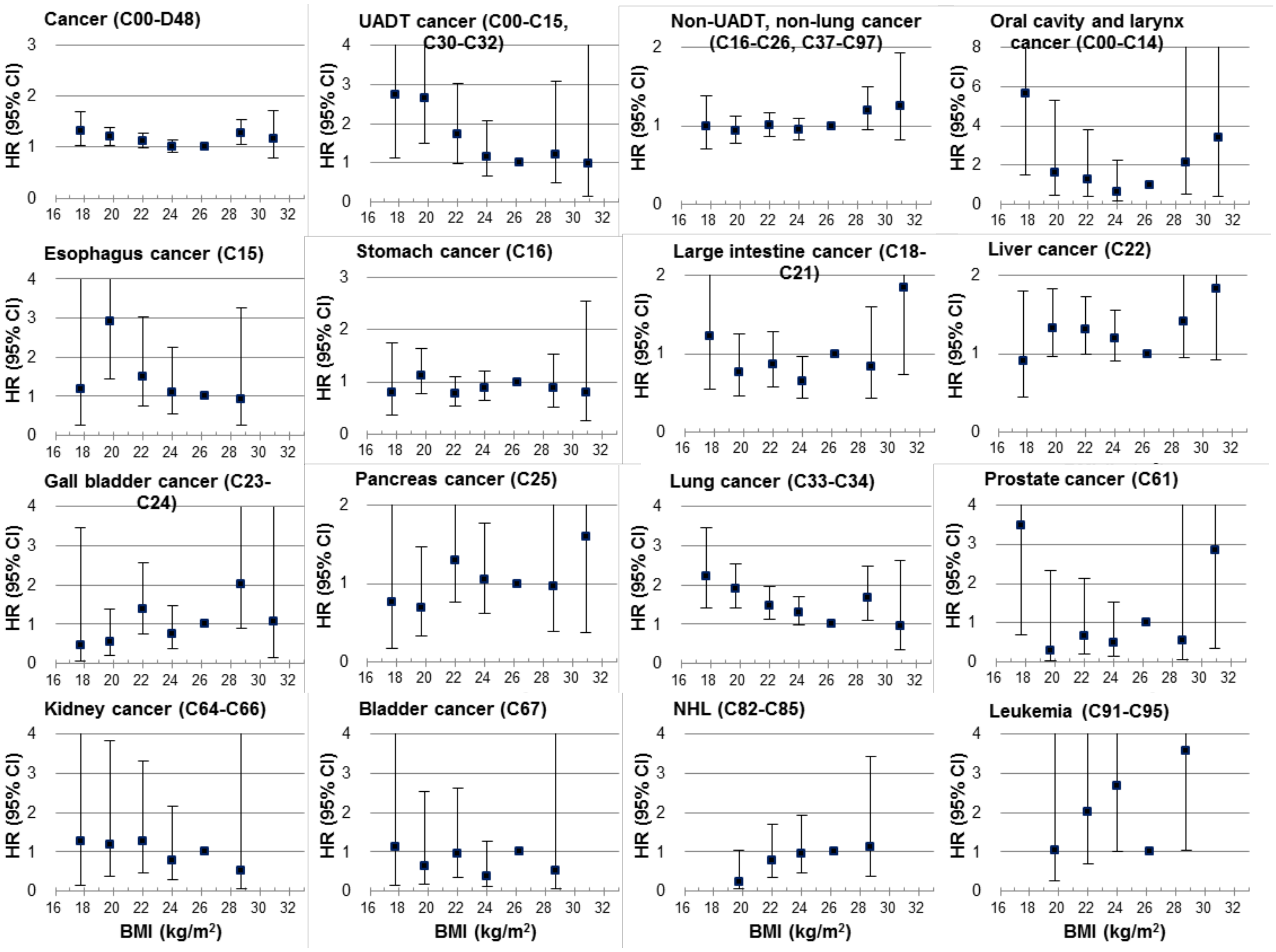

eFigure 3. Hazard ratios for cancer mortality after exclusion of the first two years of follow-up and pre-existing cancers at baseline across seven categories of BMI. The midpoint BMI was used as a representative value for each category(kg/m^2; 12-18.4, 18.5-20.9, 21-22.9, 23-24.9, 25-27.4[Reference], 27.5-29.9, 30.0-47), except for both ends of BMI categories, in which the median was used. Analyses were adjusted for age, smoking, alcohol intake, household income, and physical activity. For some causes, no death was observed in the highest or the lowest BMI categories.

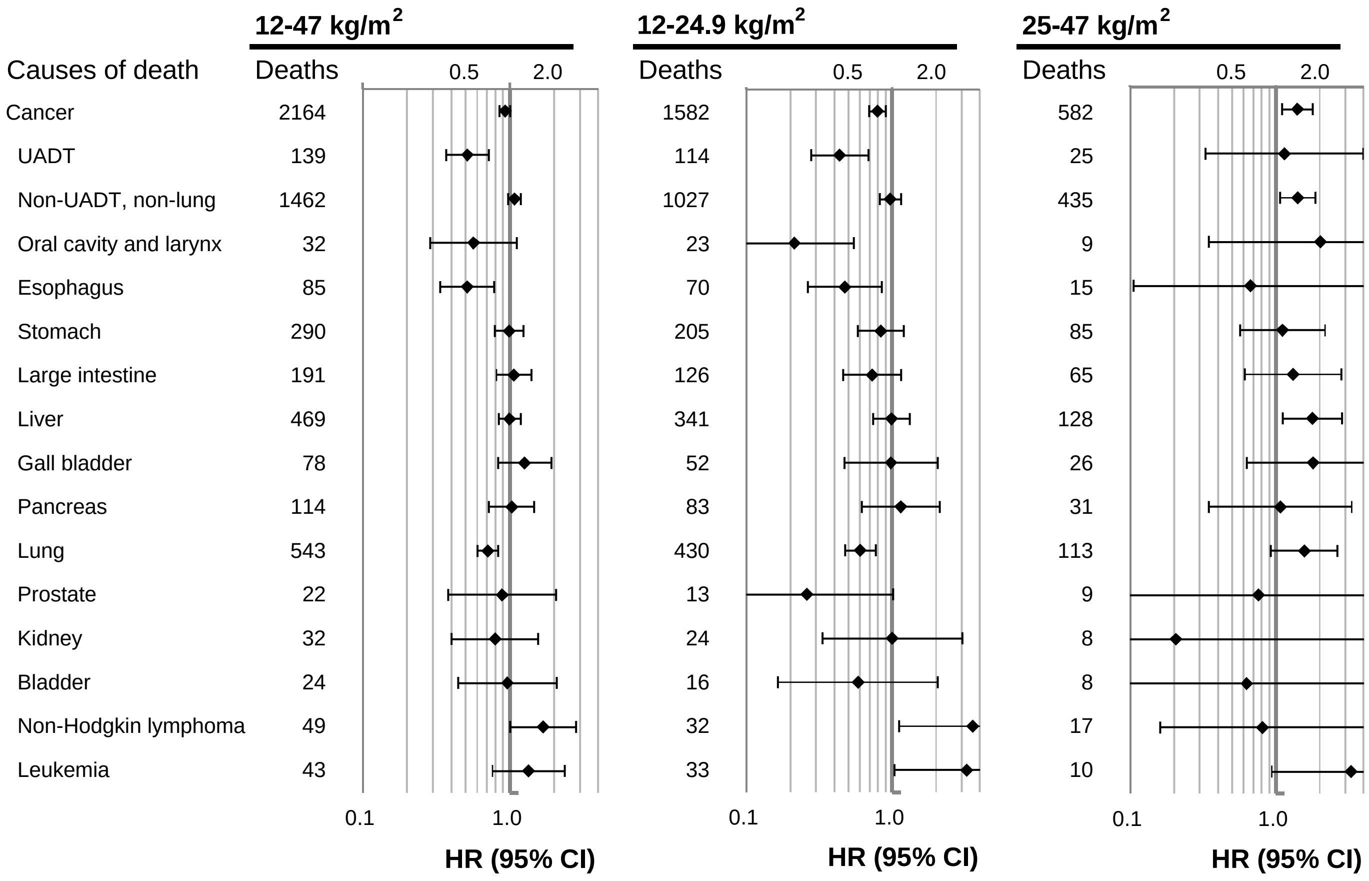

eFigure 4. Hazard ratios for cancer mortality per 5 kg/m^2 higher BMI across BMI groups after exclusion of the first two years of follow-up and pre-existing cancers at baseline. Analyses were adjusted for age, smoking, alcohol intake, household income, and physical activity. Cancers which had 10 or more cases in 12-24.9 or 25-47 kg/m^2 among all participants were included

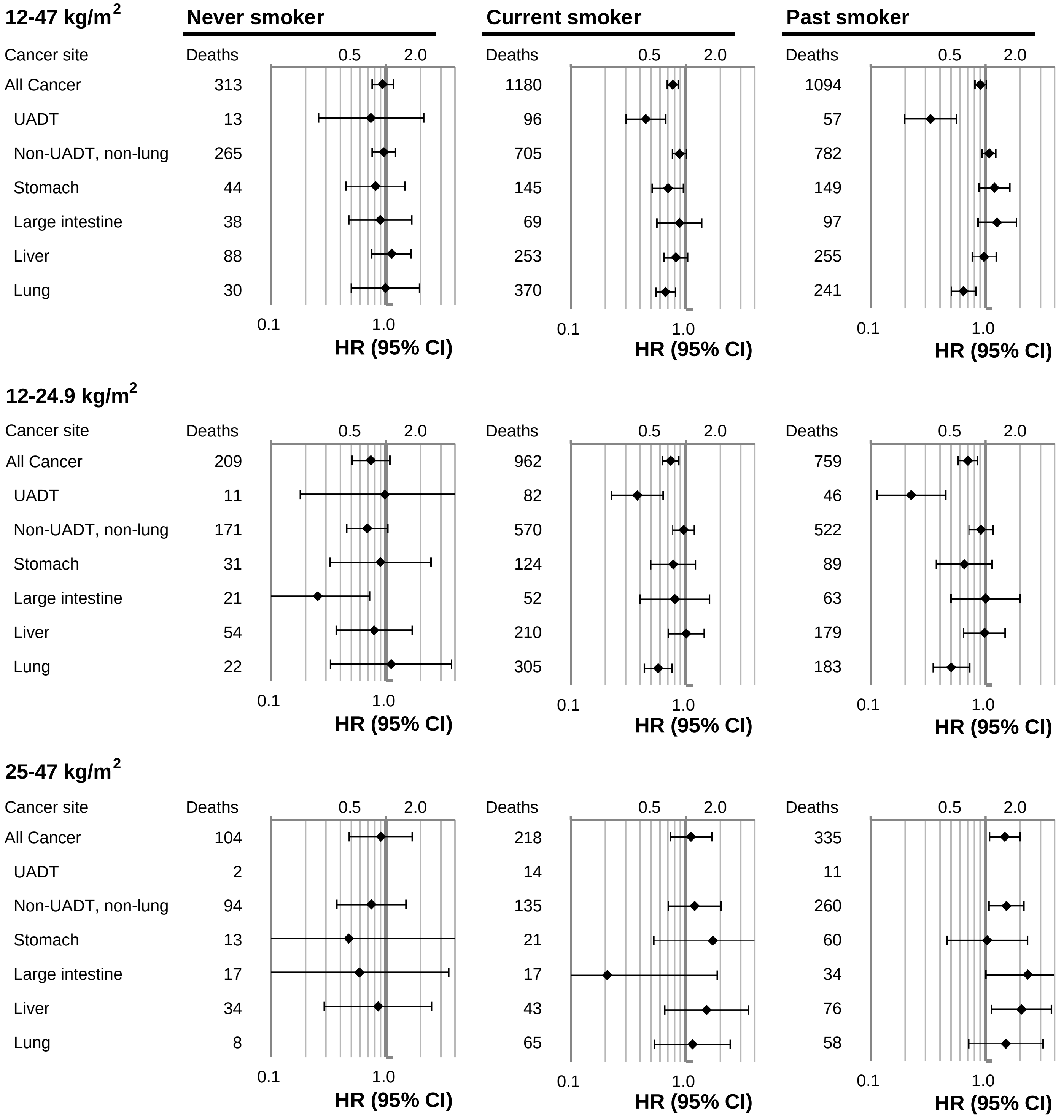

eFigure 5. Hazard ratios for selected cancer mortality per 5 kg/m2 higher BMI across BMI groups and smoking status in participants with no pre-existing cancers at baseline. Analyses were adjusted for age, smoking, alcohol intake, household income, and physical activity. Cancers which had 10 or more cases in never-smokers among all participants in 12-24.9 or 25-47 kg/m^2 were included.

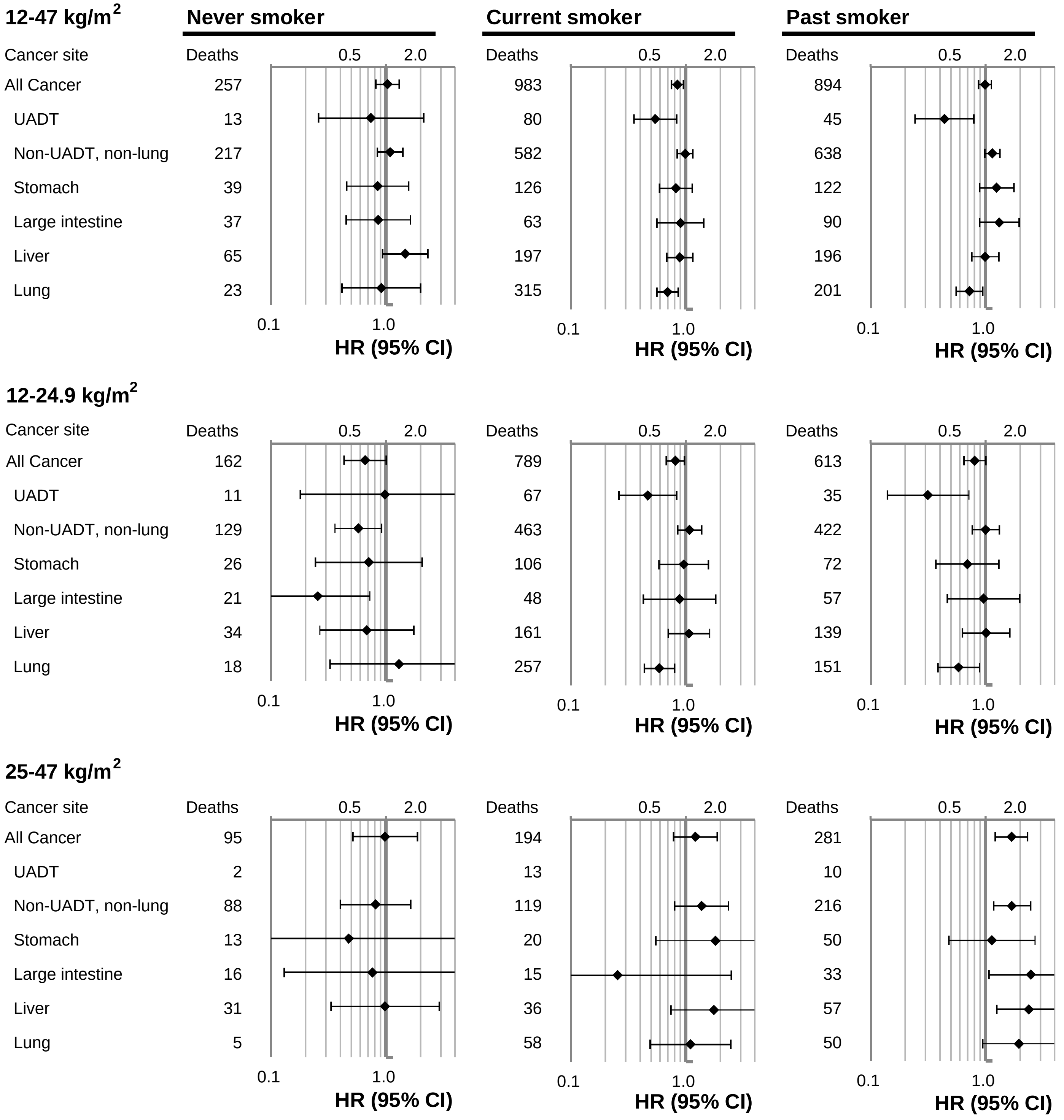

eFigure 6. Hazard ratios for selected cancer mortality per 5 kg/m2 higher BMI across BMI groups and smoking status after exclusion of the first two years of follow-up and pre-existing cancers at baseline. Analyses were adjusted for age, smoking, alcohol intake, household income, and physical activity. Cancers which had 10 or more cases in never-smokers among all participants in 12-24.9 or 25-47 kg/m^2 were included.
